# Learning ophthalmic anatomy with AI-generated visual resource: the moderating role of educational background

**DOI:** 10.3389/fmed.2026.1868565

**Published:** 2026-07-15

**Authors:** Yifan Luo, Taowei Ge, Xianglin Luo, Zhongjing Lin, Min Li, Bilian Ke

**Affiliations:** 1 Department of Ophthalmology, Renji Hospital, Shanghai Jiao Tong University School of Medicine, Shanghai, China; 2 University of Shanghai for Science and Technology, Shanghai, China; 3 Shanghai Jiao Tong University, Shanghai, China

**Keywords:** active learning, expertise reversal effect, generative artificial intelligence, ophthalmic anatomy, ophthalmology education

## Abstract

**Background:**

Declining ophthalmology teaching hours necessitate efficient instructional tools. While generative AI frequently produces structural deviations, these variations can be strategically repurposed as valuable stimuli for comparative learning. This study evaluated the effects of an AI-assisted comparative exercise versus conventional anatomical labeling on knowledge acquisition, learner satisfaction, and cognitive workload.

**Methods:**

We conducted a quasi-experimental 2 × 2 study with 121 sophomores from two universities in Shanghai, China. Following a standardized 20-min ophthalmic anatomy lecture, intact classes were assigned to either a conventional diagram-labeling task or an AI-assisted comparative exercise. The AI condition included three anatomically correct reference images paired with three expert-curated AI-generated anatomical variants, produced through a systematic expert-in-the-loop approach using Gemini 3.0 Pro. Outcomes comprised baseline and post-intervention knowledge tests, a 5-item satisfaction questionnaire, and the NASA Task Load Index.

**Results:**

After baseline adjustment and correction for planned comparisons, no statistically significant AI-versus-conventional difference in post-test knowledge scores was detected (all *p* > 0.05). Among non-medical students, the AI-assisted comparative exercise was associated with higher composite satisfaction (9.17 vs. 7.52; Holm-adjusted *p* < 0.001; *r* = 0.60) and better self-assessed performance (7.76 vs. 6.03; Holm-adjusted *p* = 0.003; *r* = 0.47), whereas composite NASA-TLX scores did not differ between AI and conventional conditions within either background group. These satisfaction and self-assessed performance benefits were not observed among medical students (all *p* > 0.05).

**Conclusion:**

A brief AI-assisted comparative exercise did not demonstrate a statistically conclusive advantage in immediate knowledge outcomes, but was associated with higher satisfaction and better self-assessed performance among non-medical students without increasing composite NASA-TLX scores. Carefully curated AI-generated anatomical variants may therefore serve as a structured adjunct for novice ophthalmic anatomy learning.

## 1 Introduction

Ophthalmology receives less teaching time than it once did. A systematic review of 52 studies across 19 countries documented a drop from an average of 92.9 course hours in the 2000s to 52.9 h in the 2020s, with North American programs among the shortest (1). Under these compressed schedules, many students graduate with limited hands-on exposure to ocular anatomy and often report low confidence in basic eye examination skills. The trend reflects a broader tension in health professions education, where expanding content competes with shrinking contact hours (2).

To optimize the use of these constrained hours, educators are progressively adopting generative artificial intelligence (AI) as a pedagogical instrument. Generative AI has already reshaped instructional methods (3–6). Large language models now answer ophthalmology subspecialty questions with variable but improving accuracy (7), and AI-generated images are being evaluated for surgical and diagnostic training (8–11). However, text-to-image models often produce minor anatomical discrepancies or hallucinations (12, 13). Emerging viewpoints propose that defective visuals can be repurposed as intended teaching stimulus, rather than being dismissed as technological failures (14, 15).

Comparison-based active learning, in which learners identify discrepancies between a target representation and an accurate reference, has a strong empirical foundation. Studies on worked examples that pair correct and flawed solutions have shown that spotting and correcting mistakes can promote deeper processing than studying correct solutions alone (16, 17). In medical education, diagnostic cases with built-in errors have improved clinical reasoning when paired with feedback (18). This rationale aligns with the desirable difficulties principle in cognitive psychology, where tasks that require more effort during learning tend to produce stronger retention and transfer over time (19, 20). When learners compare a visual depiction with an accurate reference and prior knowledge, they actively evaluate the target concept by judging whether specific features are correct or incorrect, which strengthens the underlying knowledge (21).

Nevertheless, active-learning designs that introduce productive difficulty do not yield the same benefits for all learners. The type, sequencing, and plausibility of the visual materials presented all interact with the knowledge the learner already possesses (22). Kalyuga et al. (23) referred to this interaction as the expertise reversal effect. Instructional formats that assist novices by introducing productive difficulty can become redundant or even counterproductive for more advanced learners, who already possess the scaffolding that the task provides (2). Schrader and Kalyuga (24) showed that the reversal affects emotional reactions as well as test performance, with more experienced students reporting less interest on assignments they consider unnecessary. Therefore, it remains unclear how a learner’s baseline expertise shapes their cognitive and affective responses to AI-altered anatomical images.

Although the theoretical value of comparison-based active learning is well established, its practical application using AI-generated visual inaccuracies in spatial anatomy teaching remains under-researched. In addition, it is uncertain how a learner’s previous educational experience affects the effectiveness of these technologies. To the best of our knowledge, the use of AI-generated images containing targeted anatomical variants as instructional stimuli for a structured comparison task has not yet been systematically explored in ophthalmic anatomy education. Accordingly, a quasi-experimental study was conducted to investigate whether an AI-assisted comparative exercise yields differential outcomes in knowledge acquisition, learner satisfaction, and perceived cognitive workload compared to traditional anatomical labeling practice.

## 2 Methods

### 2.1 Study design

A 2 × 2 between-subjects quasi-experimental design was utilized. The two factors were educational background (medical vs. non-medical) and instructional condition (AI-assisted comparative exercise vs. conventional labeling). Intact classes were assigned to conditions based on scheduling availability, without individual-level randomization. To minimize between-group variability, all sessions were delivered by the same instructor during the same academic term. The primary outcome was post-test knowledge performance, while the secondary outcomes were learner satisfaction and perceived cognitive workload.

### 2.2 Participants

One hundred twenty-one sophomores were recruited from two universities in Shanghai, China, during the 2025–2026 academic year. Sixty-three were medical students at Shanghai University of Medicine and Health Sciences who had completed systematic human anatomy but had not studied ophthalmic anatomy. The remaining 58 were non-medical students at the University of Shanghai for Science and Technology with no prior anatomical coursework. Within each university, intact classes were assigned to either the AI condition or conventional labeling condition, yielding four groups: medical conventional (*n* = 26), medical AI (*n* = 37), non-medical conventional (*n* = 29), and non-medical AI (*n* = 29) (Table 1). The study was approved by the Institutional Review Board of Renji Hospital, Shanghai Jiao Tong University School of Medicine (Approval No. LY2024-327-A). All participants gave written informed consent.

**Table 1:** Participant characteristics and knowledge outcomes by group.

Variable	Med + Conv (*n* = 26)	Med + AI (*n* = 37)	Medical adjusted *p*	Non-med + Conv (*n* = 29)	Non-med + AI (*n* = 29)	Non-medical adjusted *p*
Pre-test score	80.77 (17.42)	92.43 (15.88)	0.003	65.52 (22.61)	68.97 (21.77)	0.758
Pre-test at ceiling	34.60%	75.70%	–	6.90%	20.70%	–
Post-test score	73.08 (16.92)	75.14 (16.09)	0.827	58.28 (13.38)	67.24 (19.80)	0.088
Adjusted post-test score	72.62 [66.21, 79.02]	72.84 [67.03, 78.65]	0.959	60.22 [53.88, 66.56]	68.64 [62.44, 74.84]	0.108
Change score	−7.69 (24.38)	−17.30 (19.67)	0.211	−7.24 (22.02)	−1.72 (27.00)	0.566

Values are mean (standard deviation). Sample size is reported as n. Pre-test and post-test scores range from 0 to 100. Change score was calculated as post-test score minus pre-test score. Pre-test at ceiling indicates the percentage of participants achieving the maximum pre-test score of 100. Adjusted post-test scores are estimated marginal means with 95% confidence intervals from ANCOVA controlling for pre-test score. Adjusted *p*-values are Holm-adjusted *p*-values for planned AI-versus-conventional comparisons within each educational background. Med, medical; Non-med, non-medical; Conv, conventional; AI, AI-assisted comparative exercise.

### 2.3 Instructional materials

A standardized lecture module on the anatomy of the human eye was developed by a board-certified ophthalmologist. The module covers the external structures, lacrimal apparatus, extraocular muscles, three-layered structure of the eyeball (fibrous, vascular, and neural tunics), lens, vitreous body, and aqueous humor circulation. The module was evaluated for content accuracy by two senior anatomy faculty members. This lecture was delivered by the same instructor to all participants.

**Figure 1:**
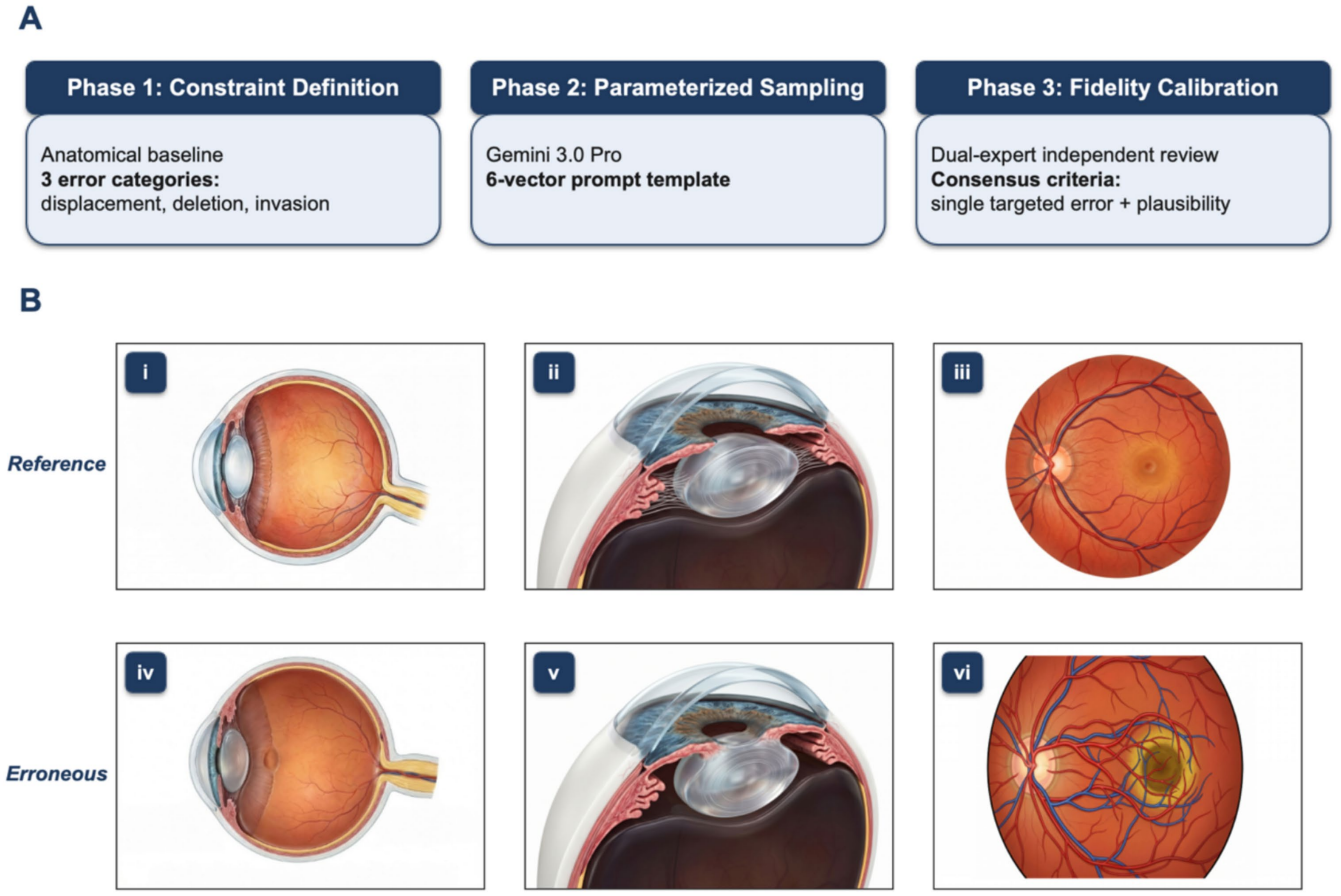
The structured Expert-in-the-Loop AI generation pipeline and representative anatomical stimuli. **(A)** The three-phase workflow includes constraint definition, parameterized sampling, and fidelity calibration. **(B)** The retained pedagogical stimulus set comprising three matched image pairs. The top row (Reference) displays anatomically correct baseline images: (i) horizontal cross-section of the eye, (ii) anterior segment with intact zonular fibers, and (iii) normal fundus preserving the foveal avascular zone (FAZ). The bottom row (Erroneous) presents the corresponding AI-generated variants with targeted structural deviations: (iv) macular displacement, (v) absence of zonular fibers, and (vi) absence of the FAZ featuring anomalous foveal vessels.

For the AI condition, images were generated via a structured three-phase Expert-in-the-Loop pipeline (Figure 1). Firstly, an ophthalmologist defined the anatomical baseline and three target deviations spanning three topological classes: structural deletion (absent lens zonular fibers), spatial displacement (abnormally located macula), and structural invasion (blood vessels within the avascular foveola). Secondly, candidate images were generated using Gemini 3.0 Pro via Google AI Studio (Google LLC, Mountain View, CA, USA) with a six-component prompt template (Supplementary Table S1). Gemini 3.0 Pro was selected because preliminary generation attempts suggested adequate prompt controllability for the targeted ocular deviations and because it could be integrated into the expert-in-the-loop workflow. Finally, two senior experts (an ophthalmologist and an anatomy faculty member) independently reviewed the candidate images. For AI-generated variants, retention required exactly one targeted anatomical deviation while preserving global anatomical plausibility. Eighteen candidate images, including anatomically correct reference images and AI-generated anatomical variants, were generated and independently screened by two experts. Six images were retained for the final instructional set, yielding a retention rate of 33.3%. Disagreements on 3 of 18 candidates were resolved through consensus discussion. Candidate images were excluded when they contained multiple unintended deviations, failed to depict the target anatomical deviation clearly, or showed globally implausible ocular anatomy. Inter-rater agreement for candidate image retention reached 83.3% with a Cohen’s kappa of 0.65, indicating substantial agreement. The production of each pedagogical image is represented as $\;I_{\mathrm{ped}}=f(A_{\text{base}},|E_{\text{target}},|\;\tau_{\text{expert}})$ , where $A_{\text{base}}$ is the baseline anatomy, $E_{\text{target}}\;$ is the targeted error, and $\;\tau_{\text{expert}}$ is the expert plausibility threshold. During the 10-min exercise, students were presented with three reference-erroneous image pairs (six images total, one anatomically correct and one deliberately altered per pair) and were asked to identify the specific anatomical deviation in each error image while consulting their lecture-based knowledge. After students first attempted to identify each deviation, the instructor provided standardized immediate feedback by indicating the targeted erroneous region, showing the corresponding anatomically correct reference image, and explaining the correct anatomical structure.

For the conventional condition, participants completed a standardized diagram-labeling exercise by identifying key structures from memory on unlabeled ocular schematics, which were adapted from a validated institutional teaching repository. Students evaluated their own performance by comparing their labels to a verified solution key after this active retrieval stage.

### 2.4 Procedure

**Figure 2:**
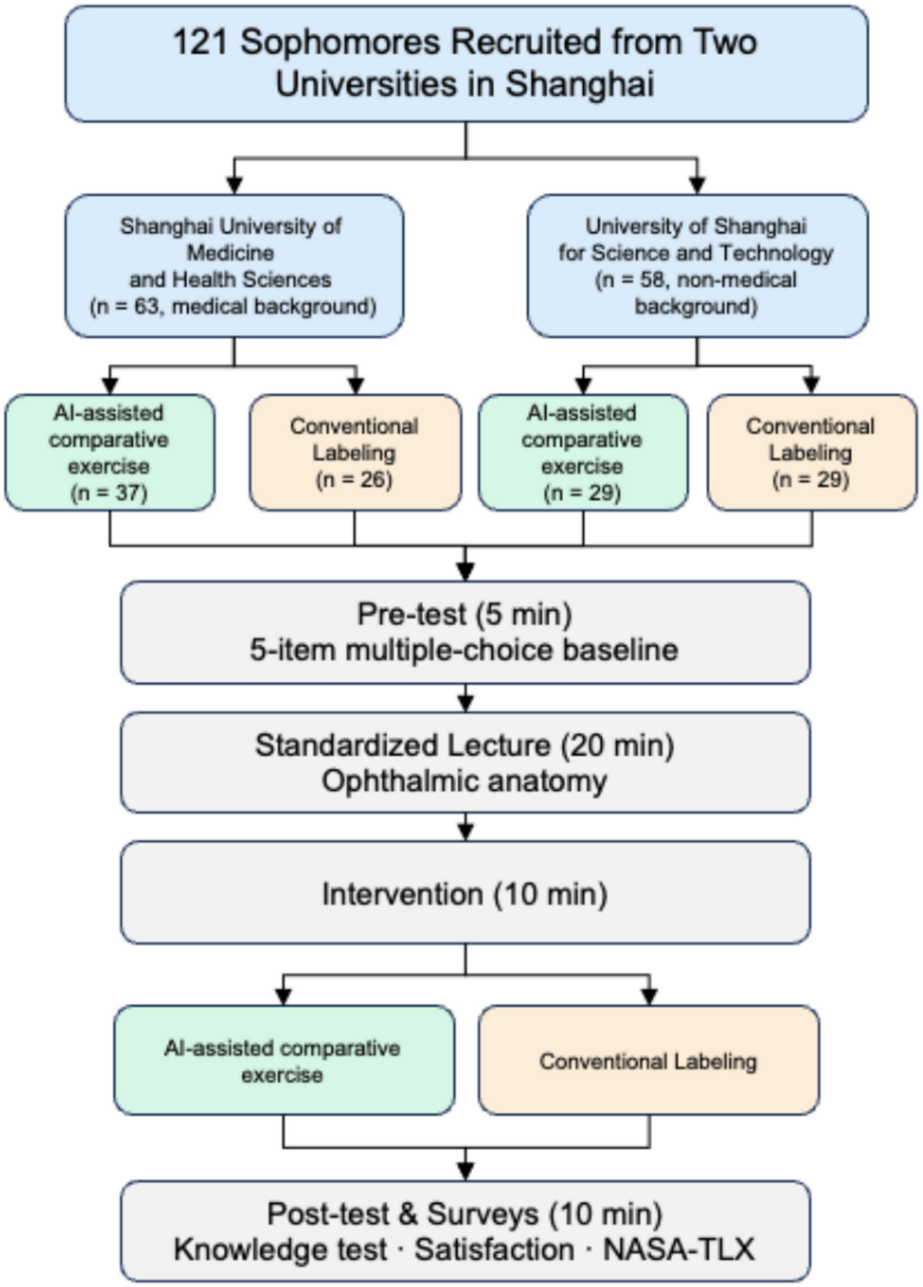
Participant flow diagram. The final dataset included 121 participants, comprising 63 students with a medical background and 58 students with a non-medical background. Participants were analyzed according to educational background and intervention condition. No outcome data were missing, and no participants were excluded from the final analysis.

The study was conducted during a single 45-min session. All participants followed the same sequence (Figure 2):

Pre-test (5 min): A 5-item multiple-choice baseline assessment.

Lecture (20 min): The standardized ophthalmic anatomy presentation.

Intervention (10 min): The assigned AI-assisted comparative exercise or conventional labeling task.

Post-test and questionnaires (10 min): A 10-item knowledge assessment comprising five multiple-choice and five fill-in items, followed by satisfaction and cognitive workload surveys. Fill-in-the-blank responses were scored using a predefined standard answer key by another professor who was blinded to instructional condition. Satisfaction and NASA-TLX questionnaires were completed anonymously; the instructor/research staff remained in the classroom for procedural supervision but did not view individual responses, and questionnaires were collected uniformly after completion.

### 2.5 Outcome measures

#### 2.5.1 Knowledge assessment

Baseline and post-intervention knowledge were assessed with a 5-item and a 10-item test comprising five multiple-choice and five fill-in items, respectively. Both tests were developed by the research team and content-validated by two ophthalmology faculty members. Scores were converted to a 0–100 scale.

#### 2.5.2 Learner satisfaction

A 5-item questionnaire was developed to assess prespecified learner-response domains relevant to this activity, including interest, perceived understanding, intellectual challenge, format preference, and self-efficacy on a 10-point scale (1 = strongly disagree, 10 = strongly agree). The arithmetic mean of the five items served as the composite satisfaction score; individual dimensions were analyzed as exploratory endpoints. The five satisfaction items showed high internal consistency (Cronbach’s alpha = 0.929).

#### 2.5.3 Cognitive workload

Perceived workload was measured with the raw (unweighted) NASA Task Load Index (NASA-TLX) (25). Participants rated six subscales (mental demand, physical demand, temporal demand, performance, effort, frustration) on an 11-point scale (0–10). The performance subscale was reverse-scored so that higher values indicated better self-assessed performance. The composite score was the arithmetic mean of all six subscales.

### 2.6 Statistical analysis

All statistical analyses were performed using R version 4.2.2 (R Foundation for Statistical Computing, Vienna, Austria), with a two-sided significance threshold of *α* = 0.05. Descriptive data are presented as mean (standard deviation, SD) and median (interquartile range, IQR). Normality was determined using the Shapiro–Wilk test. For the primary knowledge outcome, we fitted an ANCOVA model with post-test score as the dependent variable, educational background, instructional condition, and their interaction as fixed factors, and pre-test score as a covariate. Estimated marginal means and adjusted AI-versus-conventional contrasts were reported with 95% confidence intervals. The Kruskal-Wallis *H* test was used for four-group comparisons, whereas the Mann–Whitney *U* test was utilized for within-group comparisons. Post-test knowledge score was specified as the primary outcome. Composite satisfaction, composite NASA-TLX score, and the NASA-TLX self-assessed performance subscale were prespecified secondary outcomes. For each prespecified outcome, the two planned within-background AI-versus-conventional contrasts were adjusted using the Holm method. Exploratory dimension- or subscale-level analyses for satisfaction items and the remaining NASA-TLX subscales were corrected using the Benjamini-Hochberg procedure at a false discovery rate of q = 0.05. Effect sizes are reported as rank-biserial correlation coefficients (r) with 95% confidence intervals. Positive *r* values indicate higher scores in the AI condition, whereas negative *r* values indicate higher scores in the conventional condition; interpretation depends on the direction of each outcome. Absolute r values of 0.10, 0.30, and 0.50 were interpreted as small, medium, and large effects, respectively. A supplemental change-score analysis (post-test minus pre-test) was performed.

## 3 Results

### 3.1 Participant characteristics and baseline comparison

One hundred twenty-one students completed the study. Table 1 summarizes participant characteristics and knowledge outcomes by educational background and intervention condition. The pre-test scores exhibited a significant ceiling effect, especially among medical students, with 75.7% of medical students in the AI condition and 34.6% of medical students in the conventional condition achieving the maximum score of 100 points. Among non-medical students, the corresponding ceiling proportions were 20.7% in the AI condition and 6.9% in the conventional condition.

The four groups had significantly different pre-test scores [Kruskal-Wallis *H*(3) = 34.82, *p* < 0.001]. Medical students scored significantly higher than non-medical students (median 100.0 vs. 70.0; Mann–Whitney U = 2797.5, *p* < 0.001, *r* = 0.53, 95% CI [0.37, 0.68]). Within the medical group, pre-test scores were significantly higher in the AI condition compared to the conventional condition (mean 92.4 vs. 80.8; Mann–Whitney *U* = 679.0, Holm-adjusted *p* = 0.003, *r* = 0.41, 95% CI [0.16, 0.63]). However, no such discrepancy was observed among non-medical students (mean 69.0 vs. 65.5; Mann–Whitney *U* = 439.5, Holm-adjusted *p* = 0.758, *r* = 0.05, 95% CI [−0.25, 0.34]).

### 3.2 Knowledge outcomes

**Figure 3:**
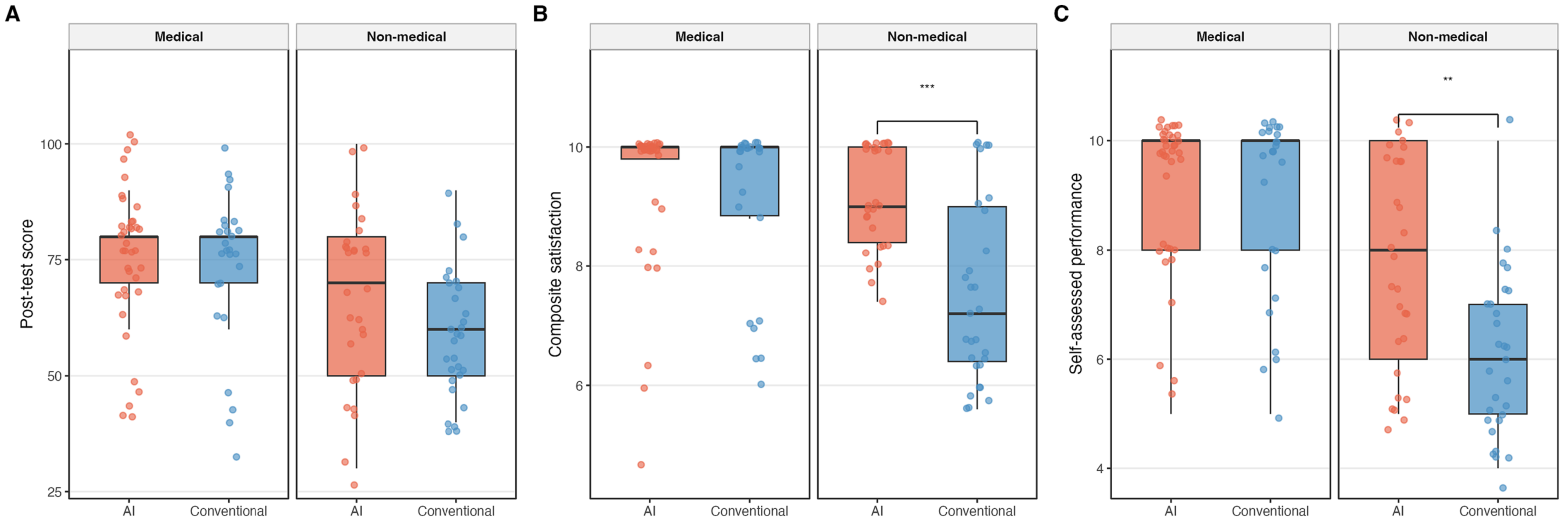
Within-group comparisons of outcomes by educational background and intervention condition. **(A)** Post-test knowledge scores (range 0–100). **(B)** Composite satisfaction, calculated as the arithmetic mean of five dimensions (interest, understanding, challenge, preference, efficacy; range 1–10). **(C)** NASA-TLX self-assessed performance subscale (range 0–10; reverse-scored so that higher values indicate better self-assessed performance). In each panel, box plots show medians (horizontal lines) and interquartile ranges, overlaid with jittered individual data points. Red, AI-assisted comparative exercise; blue, conventional labeling. Asterisks indicate statistically significant planned AI-versus-conventional comparisons within each educational background after Holm correction. **p < 0.01, ***p < 0.001.

Post-test scores differed significantly across the four groups (Kruskal-Wallis *H*(3) = 19.00, *p* < 0.001). Overall, medical students did better than non-medical students (median 80.0 vs. 60.0; Mann–Whitney *U* = 2528.0, *p* < 0.001, *r* = 0.38; Figure 3A). In the ANCOVA model, pre-test score was associated with post-test score (*F* = 4.06, *p* = 0.046, partial eta-squared = 0.034), and educational background remained significant after adjustment (*F* = 6.05, *p* = 0.015, partial eta-squared = 0.050). The main effect of intervention (*F* = 1.98, *p* = 0.162, partial eta-squared = 0.017) and the background-by-intervention interaction (*F* = 1.83, *p* = 0.179, partial eta-squared = 0.016) were not statistically significant.

Adjusted marginal means showed virtually identical post-test scores between AI and conventional conditions among medical students (AI 72.84, 95% CI [67.03, 78.65]; conventional 72.62, 95% CI [66.21, 79.02]; adjusted difference = 0.22 points, 95% CI [−8.31, 8.75], Holm-adjusted *p* = 0.959). Among non-medical students, the adjusted mean was higher in the AI condition than in the conventional condition, but the contrast was not statistically significant after Holm correction (AI 68.64, 95% CI [62.44, 74.84]; conventional 60.22, 95% CI [53.88, 66.56]; adjusted difference = 8.42 points, 95% CI [−0.15, 16.99], Holm-adjusted *p* = 0.108).

Due to the pre-test imbalance in the medical group (Section 3.1), an additional change-score analysis (post-test minus pre-test) was performed. The AI group of medical students showed a larger numerical decrease from baseline than the conventional group, but this difference was not statistically significant after Holm correction (mean change −17.3 vs. -7.7; Mann–Whitney *U* = 368.0, Holm-adjusted *p* = 0.211, *r* = −0.23, 95% CI [−0.50, 0.06]). Among non-medical students, the change scores did not differ significantly between groups (mean change −1.7 vs. −7.2; Mann–Whitney U = 457.0, Holm-adjusted *p* = 0.566, *r* = 0.09, 95% CI [−0.23, 0.38]).

### 3.3 Learner satisfaction

The scores for the composite satisfaction endpoint differed significantly across the four groups (Kruskal-Wallis *H*(3) = 28.72, *p* < 0.001). Non-medical students in the AI condition reported greater composite satisfaction than those in the conventional condition (mean 9.17 vs. 7.52; Mann–Whitney *U* = 674.5, Holm-adjusted *p* < 0.001, *r* = 0.60, 95% CI [0.35, 0.83]). In the group of medical students, composite satisfaction exhibited no significant difference between conditions (mean 9.38 vs. 9.09; U = 537.5, Holm-adjusted *p* = 0.339, *r* = 0.12, 95% CI [−0.12, 0.36]; Figure 3B).

In the exploratory dimension-level analysis (Table 2), non-medical students in the AI condition scored higher on all five satisfaction dimensions after Benjamini-Hochberg correction: interest (BH-adjusted *p* < 0.001), understanding (BH-adjusted *p* < 0.001), intellectual challenge (BH-adjusted *p* < 0.001), format preference (BH-adjusted *p* = 0.002), and self-efficacy (BH-adjusted *p* < 0.001).

**Table 2:** Satisfaction dimension scores by educational background and intervention condition.

Dimension	Med + Conv	Med + AI	Medical U	Medical adjusted p	Medical r (95% CI)	Non-med + Conv	Non-med + AI	Non-medical U	Non-medical adjusted p	Non-medical *r* (95% CI)
Composite satisfaction	9.09 (1.42)	9.38 (1.30)	537.5	0.339	0.12 [−0.12, 0.36]	7.52 (1.52)	9.17 (0.85)	674.5	<0.001	0.60 [0.35, 0.83]
Interest	9.31 (1.35)	9.57 (1.21)	519	0.700	0.08 [−0.12, 0.29]	7.28 (1.94)	9.14 (0.92)	644	<0.001	0.53 [0.26, 0.77]
Understanding	9.23 (1.50)	9.54 (1.12)	510.5	0.700	0.06 [−0.15, 0.27]	7.83 (1.51)	9.41 (0.87)	672.5	<0.001	0.60 [0.37, 0.81]
Intellectual challenge	8.92 (1.81)	9.54 (1.26)	557.5	0.700	0.16 [−0.05, 0.38]	7.76 (1.50)	9.28 (0.88)	660	<0.001	0.57 [0.33, 0.79]
Format preference	8.73 (2.11)	9.24 (1.69)	529.5	0.700	0.10 [−0.13, 0.32]	7.83 (1.69)	9.10 (1.08)	611	0.002	0.45 [0.20, 0.69]
Self-efficacy	9.27 (1.54)	9.03 (1.95)	460	0.701	−0.04 [−0.26, 0.17]	6.93 (2.14)	8.90 (1.21)	642	<0.001	0.53 [0.27, 0.76]

Values are mean (standard deviation). Within each educational background group, AI and conventional conditions were compared using Mann–Whitney U tests, with U statistics, adjusted p values, and rank-biserial correlations (r) with 95% confidence intervals reported. The composite score was the pre-specified key satisfaction analysis and was adjusted using Holm correction for planned comparisons within this outcome. Individual satisfaction dimensions were treated as exploratory outcomes and adjusted using the Benjamini-Hochberg procedure. Benjamini-Hochberg correction was applied at an FDR of q = 0.05. Med, medical; Non-med, non-medical; Conv, conventional; AI, AI-assisted comparative exercise; CI, confidence interval; U, Mann–Whitney U statistic.

No satisfaction dimension differed significantly between AI and conventional conditions among medical students after correction (all BH-adjusted *p* > 0.05).

### 3.4 Cognitive workload

Table 3 reports NASA-TLX subscale and composite scores. The composite NASA-TLX score differed across the four groups (Kruskal-Wallis *H*(3) = 14.51, *p* = 0.002), with this overall difference reflecting higher composite scores among medical than non-medical students rather than an AI-versus-conventional effect. Planned within-background comparisons showed no significant AI-versus-conventional difference among either medical students (mean 6.14 vs. 6.76; Mann–Whitney *U* = 389.0, Holm-adjusted *p* = 0.391, *r* = −0.19, 95% CI [−0.48, 0.10]) or non-medical students (mean 5.05 vs. 4.93; Mann–Whitney *U* = 362.5, Holm-adjusted *p* = 0.391, *r* = −0.14, 95% CI [−0.45, 0.18]). The self-assessed performance subscale differed strongly across the four groups (Kruskal-Wallis *H*(3) = 43.33, *p* < 0.001). In the planned within-background comparison, non-medical students in the AI condition reported higher self-assessed performance than those in the conventional condition (mean 7.76 vs. 6.03; Mann–Whitney *U* = 620.0, Holm-adjusted *p* = 0.003, *r* = 0.47, 95% CI [0.21, 0.70]), whereas no significant difference was observed among medical students (mean 9.16 vs. 8.85; Mann–Whitney *U* = 522.5, Holm-adjusted *p* = 0.495, *r* = 0.09, 95% CI [−0.17, 0.34]; Figure 3C). Exploratory analyses of the remaining NASA-TLX subscales showed no significant AI-versus-conventional differences after Benjamini-Hochberg correction.

**Table 3:** NASA task load index (NASA-TLX) subscale and composite scores by educational background and intervention condition.

Subscale	Med + Conv	Med + AI	Medical U	Medical adjusted p	Medical r (95% CI)	Non-med + Conv	Non-med + AI	Non-medical U	Non-medical adjusted p	Non-medical r (95% CI)
NASA-TLX composite	6.76 (2.46)	6.14 (2.28)	389	0.391	−0.19 [−0.48, 0.10]	4.93 (0.97)	5.05 (2.23)	362.5	0.391	−0.14 [−0.45, 0.18]
Mental demand	8.00 (3.10)	6.73 (3.16)	360	0.418	−0.25 [−0.51, 0.02]	5.79 (1.74)	5.41 (2.98)	422	0.981	0.00 [−0.29, 0.31]
Physical demand	5.00 (4.22)	4.24 (3.76)	442.5	0.583	−0.08 [−0.36, 0.20]	4.55 (1.68)	3.62 (3.18)	286	0.102	−0.32 [−0.60, −0.02]
Self-assessed performance	8.85 (1.67)	9.16 (1.38)	522.5	0.495	0.09 [−0.17, 0.34]	6.03 (1.52)	7.76 (1.99)	620	0.003	0.47 [0.21, 0.70]
Effort	8.62 (2.30)	9.19 (1.54)	518	0.583	0.08 [−0.16, 0.32]	6.90 (1.78)	6.66 (2.54)	414.5	0.981	−0.01 [−0.31, 0.27]
Frustration	4.81 (4.32)	3.76 (3.85)	427	0.583	−0.11 [−0.39, 0.17]	3.14 (2.34)	3.62 (3.82)	395	0.981	−0.06 [−0.37, 0.25]
Temporal demand	5.27 (3.86)	3.78 (3.79)	377	0.418	−0.22 [−0.49, 0.07]	3.14 (1.36)	3.24 (3.50)	321	0.232	−0.24 [−0.54, 0.08]

Values are mean (standard deviation). All subscales range from 0 to 10. The performance subscale was reverse-scored so that higher values indicate better self-assessed performance. The composite score was calculated as the arithmetic mean of all six subscales. Within each educational background group, AI and conventional conditions were compared using Mann–Whitney *U* tests, with U statistics, adjusted *p-*values, and rank-biserial correlations (r) with 95% confidence intervals reported. Adjusted *p-*values are Holm-adjusted for planned comparisons of the composite NASA-TLX score and the self-assessed performance subscale. The remaining NASA-TLX subscales were treated as exploratory outcomes and corrected using the Benjamini-Hochberg procedure at a false discovery rate of *q* = 0.05. Positive *r* values indicate higher scores in the AI condition. Med, medical; Non-med, non-medical; Conv, conventional; AI, AI-assisted comparative exercise; CI, confidence interval; NASA-TLX, National Aeronautics and Space Administration Task Load Index.

### 3.5 Effect size summary

**Figure 4:**
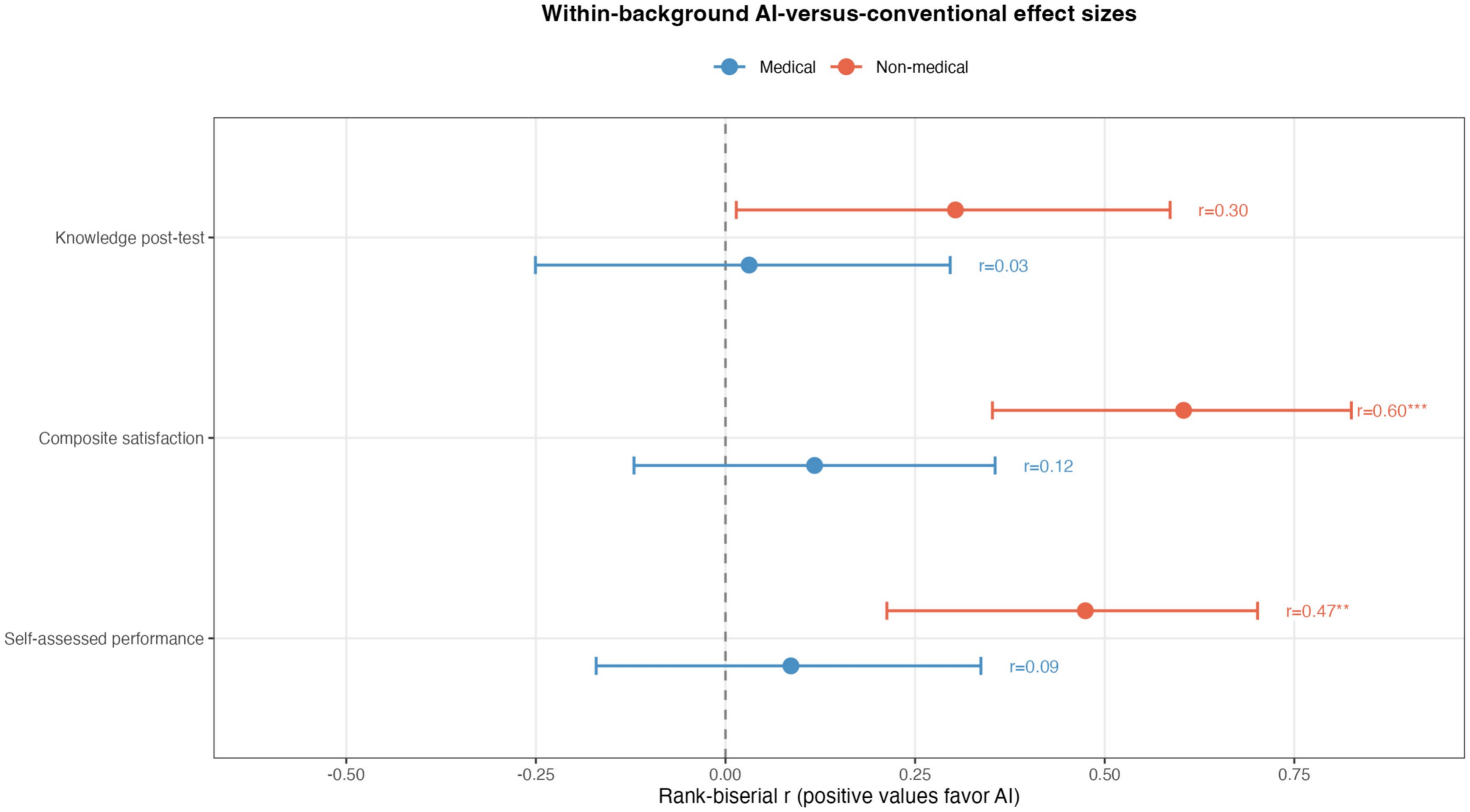
Forest plot of rank-biserial effect sizes (r) with 95% confidence intervals for within-group comparisons (AI-assisted comparative exercise vs. conventional labeling). Each row represents one outcome measure within one educational background group (six comparisons total). The dashed vertical line at zero indicates no effect; positive values indicate higher scores in the AI condition. Asterisks indicate comparisons that remained statistically significant after Holm correction. **p < 0.01, ***p < 0.001. CI, confidence interval.

Figure 4 presents a forest plot of rank-biserial effect sizes (r) with 95% confidence intervals for the within-group comparisons (AI vs. conventional). For medical students, all effect sizes were small (all |*r*| < 0.15). For non-medical students, the largest effects were observed for composite satisfaction (*r* = 0.60, Holm-adjusted *p* < 0.001) and self-assessed performance (*r* = 0.47, Holm-adjusted *p* = 0.003). The non-medical post-test comparison showed a positive medium-sized rank-biserial effect (*r* = 0.30), but this comparison did not remain statistically significant after Holm correction (Holm-adjusted *p* = 0.088), and the corresponding ANCOVA contrast was also non-significant after Holm correction (Holm-adjusted *p* = 0.108).

## 4 Discussion

This study evaluated an active-learning tool that pairs anatomically correct reference images with carefully curated AI-generated variants. Regarding cognitive outcomes, the AI-assisted condition did not show a statistically conclusive advantage in post-test knowledge scores after baseline adjustment and correction for planned comparisons. Notably, among non-medical students, the AI-assisted group reported higher satisfaction and better self-assessed performance than the conventional group, while composite NASA-TLX scores did not differ between AI-assisted and conventional conditions within either educational background group. These findings suggest that carefully curated AI-generated visual variants may enhance learner engagement and perceived learning support, particularly for novices, while their immediate effect on objective knowledge acquisition remains uncertain.

The knowledge results showed no statistically significant difference between the AI-assisted and conventional conditions after adjustment and correction. Within each educational background group, AI-assisted and conventional groups did not differ significantly in adjusted post-test knowledge scores, although the non-medical AI group showed a positive but non-significant direction after correction (all adjusted *p* > 0.05). For medical students, this finding should be interpreted in light of the high pre-test ceiling rate, particularly in the AI condition (75.7%), which may have limited the post-test’s sensitivity to detect additional learning gains in this subgroup. These findings are consistent with recent evidence in medical education, where brief technology-supported learning activities have not shown a statistically significant disadvantage in immediate knowledge outcomes relative to conventional instruction. Recent investigations into AI-generated keratitis images (9), AI-based surgical visualization systems (26), and immersive virtual reality environments (27) have consistently found no significant difference in immediate learning outcomes between technology-supported and conventional instruction. Moreover, exercises that pair accurate references with structured variants typically produce knowledge outcomes similar to those of conventional approaches (16, 17), since detectable cognitive gains usually require multi-session, feedback-driven interventions (18). Taken together, incorporating carefully curated AI-generated anatomical materials into brief learning exercises appears feasible as an adjunctive teaching strategy, but the present results do not support claims of superior or formally equivalent knowledge acquisition.

The affective and metacognitive outcomes diverged sharply based on prior anatomy training, aligning with the expertise reversal framework (23). The AI condition significantly boosted composite satisfaction (*r* = 0.60) and self-assessed performance (*r* = 0.47) exclusively among non-medical students. Conversely, medical students exhibited no such affective shift, as both instructional conditions scored near the measurement ceiling. According to expertise reversal theory, the marginal benefit of active-learning designs often diminishes once learners develop established domain schemas (28). Therefore, for the medical group the comparison-based exercise transitioned from a foundational learning challenge into a routine pattern-recognition and review task. This baseline-dependent divergence in learner affect is well-documented across diverse educational media (24, 29). Importantly, the non-medical findings also reveal a dissociation between subjective and objective outcomes. Although these students reported higher satisfaction and better self-assessed performance in the AI-assisted condition, this did not translate into a statistically conclusive improvement in knowledge acquisition. This dissociation may reflect a gap between perceived learning support and measured immediate knowledge performance: positive reactions to a novel AI-assisted activity may increase interest and confidence, but may not be sufficient to produce a detectable post-test advantage after a brief intervention (30). In addition, self-assessed performance should be interpreted cautiously, as perceived competence may not always align with objective performance. This finding aligns with recent studies in AI-assisted medical training (31–33). Therefore, AI-generated anatomical materials may be most useful for increasing motivation, perceived support, and confidence among beginners, rather than providing a universal or immediate cognitive advantage for all learners. Because the two conditions differed not only in the use of AI-generated images but also in the form of active engagement they required, these effects cannot be attributed uniquely to the AI stimuli themselves. Future factorial designs should separate visual comparison, retrieval practice, and AI-generated error stimuli as independent instructional components.

Ophthalmic anatomy may be particularly amenable to this comparison-based approach because its core structures are small, spatially dense, and visually defined, making subtle deviations difficult for novices to detect without side-by-side reference. Although AI-generated materials showed affective and self-evaluative benefits among non-medical students, their practical application requires careful instructional management. Within-background comparisons showed no significant difference in composite NASA-TLX scores between the AI-assisted and conventional conditions, suggesting that the curated comparative exercise did not increase perceived workload in this short intervention. However, this finding should not be interpreted as evidence that AI-generated anatomical images can be deployed without expert oversight. Deploying these materials safely requires rigorous expert curation (34, 35). This expert-in-the-loop process also introduces a practical implementation cost, because candidate images must be reviewed for anatomical plausibility, limited to a predefined target deviation, and paired with accurate reference images before classroom use. Recent research found that anatomical fidelity remained highly variable across different AI models and specific biological regions (36). Therefore, educators must carefully calibrate these AI-generated stimuli. The structural deviations should be subtle enough to demand genuine analytical effort. At the same time, they must remain clear enough for systematic examination. Each variant should be followed by instructor-led feedback or debriefing to prevent erroneous impressions from being reinforced. Through deliberate instructional design, educators can transform the natural variability of AI outputs into structured opportunities for active assessment.

For real classroom implementation, AI-generated anatomical variants should be embedded within a structured teaching workflow rather than used as standalone visual materials. Instructors would need training not only in prompt design and image selection, but also in recognizing common AI-generated anatomical artifacts, defining the intended target deviation, and guiding post-task debriefing. This need for faculty preparation is consistent with broader evidence that educational innovations in health professions teaching require structured faculty development, feedback, and sustained implementation support (4, 5, 37, 38). The expert-review process also creates a practical cost, because each candidate image must be screened for anatomical plausibility and pedagogical suitability before use. To reduce this burden, future implementations could develop shared expert-vetted image banks, standardized review rubrics, and semi-automated quality-control procedures. These steps may help preserve the motivational value of AI-generated variants while making their use safer and more scalable in routine ophthalmic anatomy teaching. Beyond formal medical curricula, these findings may have exploratory relevance for novice-facing science communication, but such use would require expert-vetted images, accurate references, and educator-guided explanation (39–42).

There are several limitations in this study. Firstly, the use of intact classes rather than individual randomization may have introduced unmeasured confounders and limits causal inference. The sample was limited to two Shanghai institutions, and because educational background was aligned with institution, background-related differences may also reflect institution-level teaching context. The medical AI group also had higher baseline knowledge and a marked pre-test ceiling effect. As this was a pilot classroom controlled study, we used ANCOVA and change-score analyses to address measured baseline differences. Future studies should use larger randomized designs with blinded outcome assessment to strengthen causal inference. Secondly, the intervention was short and limited to one AI model and a small expert-curated image set. The 10-min format was chosen to fit the post-lecture classroom sequence and allow completion of three matched image-pair comparisons within one scheduled session. Thus, the study assessed immediate responses rather than long-term retention or repeated-use effects. Future studies should test longer or repeated AI-assisted comparative exercises with delayed post-tests. Thirdly, the knowledge test was self-developed, and satisfaction and self-assessed performance were learner-reported outcomes. The knowledge materials were reviewed by ophthalmology faculty, and the satisfaction scale showed high internal consistency; however, further item-level validation of the knowledge test would strengthen future studies. Broader classroom implementation will also require scalable expert-review procedures to maintain anatomical accuracy while reducing the curation burden.

In conclusion, this brief AI-assisted comparative exercise did not show a clear advantage in immediate knowledge outcomes, but was associated with higher satisfaction and better self-assessed performance among non-medical students without increasing composite NASA-TLX scores. Carefully curated AI-generated anatomical variants may serve as a structured adjunct for novice ophthalmic anatomy learning, provided that expert review, accurate reference images, and instructor-led feedback are maintained. Future studies should examine whether repeated use improves long-term retention and transfer.

## Data Availability

The raw data supporting the conclusions of this article will be made available by the authors, without undue reservation.
