# Supplementary material for: Learning ophthalmic anatomy with AI-generated visual resource: the moderating role of educational background
Source: Front Med (Lausanne). 2026 Jul 15;13:1868565. doi: 10.3389/fmed.2026.1868565 (PMC13416949; doi:10.3389/fmed.2026.1868565)
Supplement: Supplementary file 1 [file Table_1.DOCX]

**Supplementary Table S1.** All images were generated using Gemini 3.0 Pro via Google AI Studio (Google LLC, Mountain View, CA, USA) with the available generation settings set to temperature = 1.0 and top-p = 0.95. No user-specified random seed was set. CFG scale and inference step number were not exposed or configurable in the Google AI Studio image-generation workflow used in this study.

| **#Image** | **① Anatomical Subject** | **② Visual Style** | **③ Spatial / Compositional Constraint** | **④ Targeted Perturbation** | **⑤ Negative Constraint** |
| --- | --- | --- | --- | --- | --- |
| (i) Reference horizontal cross-section | Healthy human eye, horizontal cross-section, superior view | Photorealistic 3D medical illustration, medical textbook aesthetic, volumetric lighting, 8k resolution | White background, aspect ratio 3:2 | Anatomically pristine and precise. Transparent cornea, deep anterior chamber, distinct iris stroma, crystalline lens suspended by fine zonules of Zinn, ciliary body, vitreous chamber, well-laminated retina, vascular choroid, sclera, optic nerve exiting slightly nasal to macula | Absolutely no text, no labels, no words, no annotations |
| (ii) Reference anterior segment | Anterior segment of the human eye, slight oblique angle (3/4 profile view) | High-resolution 3D medical illustration, crisp anatomical rendering, clinical textbook style, macro view, 8k resolution | Aspect ratio 3:2 | ANATOMICALLY CORRECT: Zonules of Zinn are perfectly intact, correctly suspending crystalline lens. Clear, normal anatomical gap between anterior surface of lens and posterior iris | Absolutely no text, no labels, no words, no annotations |
| (iii) Reference normal fundus | Healthy human retina, fundus view | Highly detailed 3D medical illustration, educational diagram, 8k | White background, aspect ratio 3:2 | ANATOMICALLY CORRECT: foveal avascular zone perfectly preserved. Normal retinal vessels originate from optic disc, arching superiorly and inferiorly, strictly AVOIDING central macula. Foveola is avascular with soft tissue shading. Macula is tiny, subtle yellowish-orange | No text, labels, words, annotations, abnormal blood vessels, overlapping disc and macula, dark spot, black circle, giant macula, oversized fovea, black hole effect, deep pit, harsh shadow, high contrast macula |
| (iv) Variant macular displacement | Human eye, horizontal cross-section, superior view | Photorealistic 3D medical illustration, medical textbook, aesthetic, volumetric lighting, 8k resolution | White background, aspect ratio 3:2 | INTENTIONAL ANATOMICAL ERROR: Spatial reversal of posterior pole. Optic Nerve exiting incorrectly on extreme Right side. Macula lutea and foveal pit located incorrectly on LEFT side | Absolutely no text, no labels, no words, no annotations |
| (v) Variant absent zonular fibers | Anterior segment of the human eye, slight oblique angle (3/4 profile view) | High-resolution 3D medical illustration, crisp anatomical rendering, clinical textbook style, macro view, 8k resolution | Aspect ratio 3:2 | INTENTIONAL SEVERE ANATOMICAL ERROR: Zonules of Zinn completely missing. Ciliary body isolated and disconnected from lens | Absolutely no text, no labels, no words, no annotations |
| (vi) Variant absent foveal avascular zone | Human retina, fundus view | Highly detailed 3D medical illustration, educational diagram, 8k | White background, aspect ratio 3:2 | INTENTIONAL SEVERE ANATOMICAL ERROR: foveal avascular zone missing. Thick red arteries and blue veins originate from optic disc on left, travel horizontally, and grow DIRECTLY OVER and ACROSS exact center of dark Macula. Foveola completely covered by dense vascular network | Absolutely no text, no labels, no words, no annotations |
